# Genetic assessment of a summer chum salmon metapopulation in recovery

**DOI:** 10.1111/eva.12118

**Published:** 2013-11-25

**Authors:** Maureen P Small, Thom H Johnson, Cherril Bowman, Edith Martinez

**Affiliations:** 1 Washington Department of Fish and Wildlife, Molecular Genetics LaboratoryOlympia, WA, USA; 2 Point No Point Treaty CouncilKingston, WA, USA

**Keywords:** conservation, hatchery impacts, microsatellites, population structure, recovery, salmonids, supplementation

## Abstract

Programs to rebuild imperiled wild fish populations often include hatchery-born fish derived from wild populations to supplement natural spawner abundance. These programs require monitoring to determine their demographic, biological, and genetic effects. In 1990s in Washington State, the Summer Chum Salmon Conservation Initiative developed a recovery program for the threatened Hood Canal summer chum salmon Evolutionarily Significant Unit (ESU) (the metapopulation) that used in-river spawners (wild fish) for each respective supplementation broodstock in six tributaries. Returning spawners (wild-born and hatchery-born) composed subsequent broodstocks, and tributary-specific supplementation was limited to three generations. We assessed impacts of the programs on neutral genetic diversity in this metapopulation using 16 microsatellite loci and a thirty-year dataset spanning before and after supplementation, roughly eight generations. Following supplementation, differentiation among subpopulations decreased (but not significantly) and isolation by distance patterns remained unchanged. There was no decline in genetic diversity in wild-born fish, but hatchery-born fish sampled in the same spawning areas had significantly lower genetic diversity and unequal family representation. Despite potential for negative effects from supplementation programs, few were detected in wild-born fish. We hypothesize that chum salmon natural history makes them less vulnerable to negative impacts from hatchery supplementation.

## Introduction

In the Pacific Northwest of the United States, fisheries managers are developing strategies to conserve and restore native biodiversity in Pacific salmonids. After decades of harvest beyond sustainable rates and loss of spawning and rearing habitats, over 20% of native salmon populations are imperiled or extinct (Augerot and Foley 2005). Salmon hatcheries have often been employed to mitigate losses of native fish, both as means to support native populations and to support harvest opportunities. Traditional hatcheries developed broodstocks whose progeny were released into nonancestral streams (i.e., nonlocal broodstocks); rearing and release practices promoted adaptation to hatchery environments such that nonlocal, hatchery-adapted hatchery fish fared poorly under natural conditions and potentially brought maladaptive traits into wild populations (Tessier et al. 1997; Hansen et al. 2000; McClure et al. 2008; Naish et al. 2008). Currently, impacts from hatchery fish are monitored by assessing relative fitness, genetic diversity, and effective population size (*N*_e_) in supported populations (Wang and Ryman 2001; Hare et al. 2011). Recent studies have demonstrated lower fitness in populations receiving hatchery fish from traditional hatcheries, compared with local, wild populations (reviewed in Berejikian and Ford 2004; Araki et al. 2008; Christie et al. 2012). Studies suggested that hatchery fish with lower fitness or maladapted traits depressed the productivity of wild stocks with which they interbreed (Lynch and O'Hely 2001; Araki et al. 2007a, 2008). Further, introgression by common farmed broodstock into wild populations may decrease genetic distinction among wild populations (Hansen et al. 2009; Glover et al. 2012).

With concerns over the role of hatchery programs for restoring wild populations, supplementation hatchery programs were designed to decrease negative hatchery impacts. Supplementation hatcheries aim to temporarily increase spawner census size to conserve genetic resources and boost natural population abundance of imperiled wild populations while minimizing genetic and ecological risks commonly associated with traditional hatchery practices (Ford 2002; Goodman 2004). Supplementation programs developed to meet conservation objectives bring local, natural-origin adults, juveniles, or eggs into a hatchery to initiate the program for a specific tributary (Berejikian et al. 2008; Small et al. 2009) and release the hatchery-born juveniles into their specific tributary. When adult fish return to the supplementation program tributary, hatchery-and wild-born spawners intermix in the natural spawning area, and subsequent hatchery broodstocks for each target tributary are a mix of these hatchery-and wild-born spawners returning to their tributary. With supplementation hatcheries, it is hoped that increasing spawner abundance with local-origin supplementation fish will have positive effects of maintaining genetic diversity, increasing *N*_e_, and supporting persistence and adaptive potential of targeted populations (Wang and Ryman 2001; Hedrick 2005; Berejikian et al. 2008). Supplementation programs have met with varying success because hatchery practices may counter intended benefits through high variance in hatchery family sizes and unequal sex ratios, which can decrease *N*_e_ and reduce genetic diversity of supplemented populations (Ryman–Laikre effects, Ryman and Laikre 1991; see Christie et al. 2012). Some evidence suggests lower reproductive success for natural-origin steelhead (*Oncorhynchus mykiss* Walbaum) whose parents arose from a supplementation-style hatchery in comparison with wild-origin steelhead with no hatchery ancestry (Araki et al. 2007b, 2008; Christie et al. 2012). Further, Chilcote et al. (2011) suggested that these hatchery impacts on wild salmon productivity were also found in coho salmon (*Oncorhynchus kisutch* Walbaum) and Chinook salmon (*Oncorhynchus tshawytscha* Walbaum) and occurred regardless of number of generations in the hatchery or the origin (local or exotic) of the hatchery broodstock. However, Sharma et al. (2006) observed increased productivity in supplemented wild coho salmon and Hess et al. (2012) found higher abundance and no fitness loss in supplemented wild Chinook salmon.

Although chum salmon (*Onchorhynchus keta* Walbaum) have a typical anadromous Pacific salmon life history, negative impacts from a supplementation hatchery program may be lower for chum salmon than for other salmonids. Chum salmon juveniles migrate to estuaries within days of emergence (Johnson et al. 1997), and hatchery chum salmon are typically released as fry after 2–3 months of rearing (chum salmon juveniles in this supplementation program were released into their target streams within 75 days of hatching). Thus, in contrast to other species, hatchery-born chum salmon spend minimal time under the selective forces within an artificial rearing environment and impacts might be primarily through broodstock selection, lack of mate choice, and unequal success of hatchery families. In this study, we explore impacts of supplementation programs in chum salmon by examining changes in genetic diversity and effective population sizes in a thirty-year time series starting in the 1970s that includes collections spanning the time from before, during, and after supplementation. Hatchery supplementation and reintroductions began in the 1990s for summer chum salmon in Hood Canal (HC) and Strait of Juan de Fuca (SJF) in Washington State in response to declines and extinctions (see Methods for details).

## Materials and methods

### Chum salmon life history

Chum salmon spawn mainly in lower tributary reaches up to barrier falls in coastal tributaries along the northern periphery of the Pacific Ocean, as far south as Japan and Oregon. Similar to other Pacific salmon, chum salmon are semelparous and return to spawn generally after 3 or 4 years in salt water, but returns after 2 or 5 years are not uncommon. In contrast to other Pacific salmon (except pink salmon), chum salmon juveniles spend little time in freshwater, migrating downstream to estuaries and salt water a few weeks after emergence. Chum salmon stray at similar rates to other Pacific salmon (Johnson et al. 1997), but high spawner densities may increase stray rates (reviewed in Quinn 1993).

### Hood Canal summer chum salmon

**Figure 1:**
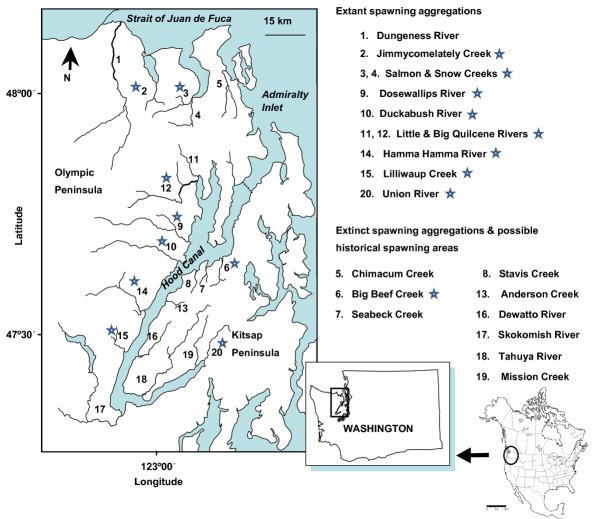
Map of Hood Canal, Strait of Juan de Fuca and portions of Puget Sound. Rivers and streams are numbered and asterisks mark tributaries in the study. At right are listed the tributary names associated with numbers and the status of subpopulations in the tributaries. Map was modified from Sands et al. (2009).

Within the Puget Sound region in Washington State, summer-run chum salmon in HC and SJF (Fig. 1) are genetically and ecologically distinct from fall-run chum salmon in the region (Phelps et al. 1994; Tynan 1997) and are designated a separate ESU (Johnson et al. 1997). Due to population declines and extinctions, the HC summer chum salmon ESU was listed as threatened under the Endangered Species Act in 1999 (http://www.nwr.noaa.gov/ESA-Salmon-Listings/Salmon-Populations/Chum/Chum-Status-Reviews.cfm). A recovery program started in the early 1990s, prior to the ESA listing, enacted crucial harvest reductions and supplementation programs. In 2000, state and tribal co-managers completed the Summer Chum Salmon Conservation Initiative (SCSCI; WDFW and PNPTT 2000), a recovery plan that formalized and expanded recovery efforts. Two independent summer chum salmon populations were designated as ESU recovery units: HC and SJF; both consisted of multiple subpopulations in rivers draining to those two marine basins and both were monitored for status relative to recovery goals. There had been small releases of hatchery summer chum salmon juveniles in various HC tributaries before 1938 (Johnson et al. 1997), but the recovery program was the first directed hatchery intervention in most tributaries.

As part of efforts to restore wild subpopulations of summer chum salmon, supplementation hatchery programs were initiated for three subpopulations (Lilliwaup, Quilcene, and Salmon) in 1992 and for three more subpopulations (Hamma Hamma, Jimmycomelately, and Union) in 1997, 1999, and 2000, respectively (each supplementation program used wild-origin broodstock collected from its respective tributary). Summer chum salmon from three streams with supplementation programs (Quilcene, Salmon, and Union) were also the donor stocks for programs used to reintroduce summer chum salmon in one tributary each (Big Beef, Chimacum, and Tahuya, respectively) where native populations had gone extinct. Two subpopulations, Dosewallips and Duckabush, were not deliberately supplemented. Supplementation programs were designed to reduce extinction risk and to speed recovery and recolonization while minimizing risks of deleterious genetic, ecological, and demographic effects to supplemented and unsupplemented subpopulations (WDFW and PNPTT 2000; Tynan et al. 2003). Supplementation hatcheries were scheduled to run for a maximum of three generations (12 years), and number of fry released into each tributary was based on available habitat. To best represent the demographics of each local donor population and to maintain existing genetic diversity, programs followed protocols (Schroder and Ames 2004) in which supplementation broodstocks for each tributary were collected from local spawners randomly in proportion to the timing, weekly abundance, and duration of the total return from the same tributary where hatchery-propagated fish were to be released (each tributary had its own supplementation program all with the goal of equal sex ratios and using no more than 50% of in-river spawners; see Table SI in Supplementary Information for broodstock details for each program). Matings employed partial factorial designs (Campton 2004; Busack and Knudsen 2007) to maximize genotypic diversity and effective subpopulation size (Waples and Do 1994; Withler and Beacham 1994). All hatchery fish were mass-marked either externally (fin-clipped, Quilcene program) or internally [all other programs received unique hatchery-specific otolith banding pattern marks (see Volk et al. 1987)]. Marks identified hatchery fish back to specific hatchery of origin from observed fin-clips or from otoliths collected from carcasses during annual spawner surveys (WDFW and PNPTT 2000; WDFW and PNPTC 2007). Because most hatchery fish were marked internally, after supplementation fish began to return, broodstock selected from returning spawners included unknown numbers of hatchery-born fish that were identified post-mortem. All supplementation program releases were mass-marked beginning with brood year 1997 (and chum salmon generally return after 3 and 4 years), and from 2001 onward managers identified hatchery-born spawners from internal and external marks (Table 2). Although we have no direct measure of the reproductive success for hatchery-born spawners, the proportion of hatchery-born spawners was an indication of maximum expected hatchery influence.

### Collections

Collections consisted of summer chum salmon from HC and SJF subpopulations (Fig. 1; Table 1). Tissue samples for this study were collected from spawners in seven rivers and creeks in HC and two creeks in SJF from 1978 through 2009 (Table 1). Samples collected prior to initiating supplementation programs were termed ‘original’ and samples collected after supplementation fish returned were divided by birth location, ‘hatchery’ or ‘wild’. A few collections included samples from more than 1 year because there were too few samples per individual year (Table 1). The following summer chum salmon subpopulations had supplementation programs: Union, Lilliwaup, Hamma Hamma, Big Quilcene, Salmon, and Jimmycomelately (see Table 2 for numbers of hatchery-born fry planted per year and see Table S2 in Supplementary Information for years when hatchery-born spawners returned). Although Big Beef Creek summer chum salmon were extirpated by 1990, we analyzed tissues (archived scales) collected in the creek prior to extirpation (1978, 1979) and after reintroduction with Quilcene stock (2004). Summer chum salmon in Dosewallips and Duckabush rivers (unsupplemented) were monitored for comparisons with supplemented subpopulations and to document and identify strays from supplementation programs. Spawners from all HC and SJF summer chum salmon subpopulations were censused annually for supplementation hatchery-and wild-born spawners in each river (post-mortem otolith analyses identified hatchery-born spawners and their specific supplementation programs). Marked otoliths or fin-clip ratios from spawner carcasses estimated the number and proportion of hatchery-born spawners (Table 2), and scales were collected to age spawners as part of biological data for recovery monitoring (WDFW and PNPTC 2007).

**Table 1:** Genetic statistics for summer chum salmon collections

							Linkage (120 pairs)	
Region	Tributary	Year	Name	*N*	*F*_IS_	*P* value	5%	1%
HC	Big Beef	1978	78BigB_O	44	−0.007	0.6526	8	3
HC	Big Beef	1979	79BigB_O	43	0.045	0.0066	9	3
HC	Big Beef	2004	04BigB_H	45	−0.003	0.5768	17	4
HC	Union	2000	00Union_O	54	0.016	0.1570	11	2
HC	Union	2003	03Union_W	32	−0.015	0.7638	5	0
HC	Union	2003	03Union_H	30	0.013	0.2706	21	9
HC	Union	2004	04Union_H	43	0.014	0.2255	28	9
HC	Union	2008	08Union_W	47	−0.022	0.9165	10	3
HC	Lilliwaup	1985	85Lilli_O	23	−0.037	0.9450	11	6
HC	Lilliwaup	1992	92Lilli_O	46	0.007	0.3291	15	3
HC	Lilliwaup	1997–99	97_99Lilli_W	35	−0.017	0.8032	18	7
HC	Lilliwaup	2000–01	00_01Lilli_W	35	0.019	0.1504	17	3
HC	Lilliwaup	2001	01Lilli_H	26	−0.006	0.5915	7	1
HC	Lilliwaup	2002	02Lilli_H	47	−0.020	0.8725	96	69
HC	Lilliwaup	2003–04	03_04Lilli_H	53	−0.006	0.6558	84	38
HC	Lilliwaup	2005–06	05_06Lilli_W	29	−0.033	0.9512	26	9
HC	Lilliwaup	2005	05Lilli_H	60	0.000	0.5112	16	2
HC	Lilliwaup	2006	06Lilli_H	55	0.022	0.0698	11	2
HC	Lilliwaup	2008	08Lilli_H	42	0.007	0.3315	12	3
HC	Lilliwaup	2009	09Lilli_H	29	0.025	0.1052	20	4
HC	Hamma Hamma	1999	99Hamma_O	34	0.033	0.0399	22	4
HC	Hamma Hamma	2001	01Hamma_W	53	0.001	0.4666	8	2
HC	Hamma Hamma	2003	03Hamma_W	40	−0.013	0.7611	11	2
HC	Hamma Hamma	2001–03	01_03Hamma_H	27	0.018	0.1830	10	4
HC	Hamma Hamma	2008	08Hamma_W	41	0.000	0.4835	10	1
HC	Dosewallips	1992	92Dose_O	48	−0.012	0.7837	6	2
HC	Dosewallips	2000	*00Dose_W	53	0.029	0.0369	11	2
HC	Dosewallips	2003	*03Dose_W	46	0.048	0.0015	6	3
HC	Dosewallips	2009	*09Dose_W	33	−0.011	0.7137	7	0
HC	Duckabush	1986	86Duck_O	57	0.014	0.1713	12	4
HC	Duckabush	1992	92Duck_O	46	−0.007	0.6837	9	2
HC	Duckabush	2000	*00Duck_W	46	0.037	0.0141	13	2
HC	Duckabush	2003	*03Duck_W	47	0.018	0.1352	9	3
HC	Duckabush	2009	*09Duck_W	34	0.018	0.1637	10	2
HC	Quilcene	1992	92Quil_O	50	−0.012	0.7824	3	1
HC	Quilcene	2008–09	08_09Quil_W	45	0.009	0.2346	12	4
SJF	Salmon	1986	86Salmon_O	42	0.024	0.6322	14	4
SJF	Salmon	2000	00Salmon_W	25	0.027	0.0938	12	4
SJF	Salmon	2000	00Salmon_H	32	0.003	0.1133	19	3
SJF	Salmon	2003–05	03_05Salmon_W	34	0.005	0.4324	17	10
SJF	Salmon	2003	03Salmon_H	27	0.018	0.4063	21	9
SJF	Salmon	2004–05	04_05Salmon_H	24	0.131	0.2010	4	0
SJF	Salmon	2008	08Salmon_W	25	−**0.018**	0.0000	16	2
SJF	Salmon	2009	09Salmon_W	23	0.014	0.7968	10	3
SJF	Jimmycomelately	1986	86JCL_O	62	−0.035	0.2754	41	12
SJF	Jimmycomelately	1998–99	98_99JCL_O	16	−0.019	0.9910	16	5
SJF	Jimmycomelately	2000	00JCL_O	38	0.019	0.7256	60	28
SJF	Jimmycomelately	2001	01JCL_W	58	0.009	0.1688	45	22
SJF	Jimmycomelately	2001–04	03_04JCL_H	75	−0.017	0.2792	80	49
SJF	Jimmycomelately	2008–09	08_09JCL_W	33	0.001	0.8896	11	3
SJF	Jimmycomelately	2008	08JCL_H	20	−0.028	0.4732	12	5
SJF	Jimmycomelately	2009	09JCL_H	34	−0.042	0.8736	19	6

Regions are abbreviated: Hood Canal = ‘HC’, Strait of Juan de Fuca = ‘SJF’. Name abbreviations used throughout document are in ‘Name’ column. Name includes collection category (O = original prior to supplementation program, H = Hatchery-origin, and W = Wild-origin during or after time of hatchery supplementation), and

* indicates collections from tributaries that were not deliberately supplemented. Statistics include the Hardy–Weinberg equilibrium value expressed by *F*_IS_, and its associated *P* value (underlined *F*_IS_ values significant before Bonferroni corrections, and bold value was significant after Bonferroni corrections), the number of locus pairs (out of 120 pairs) in linkage disequilibria at the 5% and 1% level (values in gray boxes were >5% of total pairs). Other genetic statistics, gene diversity and allelic richness, are presented in Table 4.

**Table 2:** Number of hatchery-origin fry (for brood year) released into supplemented tributaries (fry were offspring of spawners collected in their tributary the previous fall and spawned in hatchery) and summer chum salmon escapements (wild-origin ‘wild’ and supplementation hatchery-origin ‘HOS’ spawners arriving at spawning grounds) into tributaries in Hood Canal (HC) and Strait of Juan de Fuca (SJF) from 1990 through 2011. Data are from WDFW and WWTIT (2002), WDFW and PNPTT (2007), WDFW Hatchery planting database, and FishBooks (Are Strom and Kelly Henderson, WDFW)

	Union			Lilliwaup			Dosewallips			Duckabush			Hamma Hamma			Quilcene		
HC	Escapement			Escapement			Escapement			Escapement			Escapement			Escapement		
Year	Wild	HOS	fry	Wild	HOS	fry	Wild	HOS	fry	Wild	HOS	fry	Wild	HOS	fry	Wild	HOS	fry
1990	275			2			8			42			90			6		
1991	208			30			250			102			71			50		
1992	140			99		20 000	655			617			123			743		216 441
1993	251			77		12 000	105			105			69			148		24 784
1994	738			111		15 000	225			263			370			722		343 550
1995	721			79		0	2787			825			476			3057	1517	441 167
1996	494			76		15 000	6976			2650			774			7805	1710	612 598
1997	410			27		14 200	47			475			111		12 000	5231	2672	340 744
1998	223			24		17 200	336			226			127		2800	1595	1458	343 530
1999	159			13		17 400	351			92			255		51 600	1597	1640	181 711
2000	744		75 876	22		14 800	1260			464			229		55 400	3115	2783	414 353
2001	1491	0	73 472	41	51	38 000	757	233		662	280		1155	72	49 500	3048	3325	351 709
2002	872	0	82 636	36	822	96 000	1313	314		355	175		1050	1278	61 000	3 211	1276	272 017
2003	7906	4010	35 343	27	326	103 913	6510	556		1600	269		536	318	75 356	10 740	1993	92 559
2004	3598	2378		136	881	99 500	10 284	1265		7850	787		2409	282	57 000	35 838	2315	
2005	704	1283		259	790	106 466	2496	162		752	69		1185	226	117 837	5920	838	
2006	1667	1170		426	1189	88 800	2457	120		2964	171		2707	358	151 550	10 881	995	
2007	1889	78		153	372	0	1462	6		1270	24		1416	73	48 530	2479	47	
2008	1043	87		147	489	68 810	3828	102		2517	151		1371	256	208 450	3861	0	
2009	597	14		60	186	140 210	1093	34		2499	160		591	72		1492	0	
2010	897	0		188	50	139 816	2521	0		4110	0		1370	101		2073	NA	
2011	280	16		75	36	NA	1130	0		1506	0		685	87		2580	0	

	Salmon			JCL		
SJF	Escapement			Escapement		
Year	Wild	HOS	fry	Wild	HOS	fry
1990	245			63		
1991	172			125		
1992	433		19 200	616		
1993	452		44 000	110		
1994	161		2 000	15		
1995	591		38 808	223		
1996	894		62 000	30		
1997	768	66	71 821	61		
1998	605	529	67 832	98		
1999	132	367	34 680	7		3 880
2000	439	407	90 435	55		25 900
2001	1168	1470	92 415	251	9	54 515
2002	3745	1772	117 797	2	40	20 887
2003	3785	1866	88 610	68	378	50 307
2004	4103	1918		613	1049	76 982
2005	3857	2285		493	817	57 300
2006	4326	568		345	380	79 428
2007	1243	31		465	185	73 840
2008	1544	24		573	481	88 766
2009	1218	19		526	2102	92 200
2010	2740	0		739	3288	85 630
2011	2268	11		814	1597	

### Genotyping

We genotyped fish at 16 microsatellite loci [*Oke*-3 (Buchholz et al. 2001), *Oki*-1(Smith et al. 1998), *Omy*-1011 (Spies et al. 2005), *One*-101, *One*-102, *One*-106, *One*-108, *One*-111, *One*-114 (Olsen et al. 2000), *One*-18 (Scribner et al. 1996), *Ots*-1, *Ots*-2M, *Ots*-3M (Banks et al. 1999), *Ots*-103 (Small et al. 1998), *Ots*-G311 (Williamson et al. 2002), and *Ssa*-419 (Cairney et al. 2000)] for 2057 individuals from 43 collections (Table 1). DNA was extracted using silica membranes following the manufacturer's instructions (Macherey-Nagel). Microsatellite loci were amplified in six multiplexes, and alleles were scored by two researchers prior to export for analyses (see Small et al. 2009 for details of PCR, scoring, and binning).

### Statistical tests

Genetic statistics were calculated to examine whether collections met expectations of random sampling and to identify any problems with loci such as disequilibrium from large-allele dropout, null alleles, or scoring problems. Statistics assessed differences in genetic diversity between original samples, supplementation program samples (hatchery-born spawners and wild-born spawners collected in the same spawning areas), and samples from unsupplemented tributaries and evaluated changes following supplementation using tests described below. The software COLONY2.0.0.1 (Wang 2008) calculated full-sibling family structure that might contribute to Ryman–Laikre effects through unequal representation of hatchery families among spawners. Samples were tested for departures from Hardy–Weinberg equilibrium (HWE) at each locus and across all loci using FSTAT 2.9.3 (Nei 1987; Goudet 2001) with 1000 permutations. Departure from HWE can be an indication that samples contain family groups, or a strong year class, or more than one subpopulation, or related parents in the previous generation, or locus problems such as null alleles or selection. We tested whether genotypes at each locus were independent with the linkage disequilibrium permutation test in GENETIX 4.03 (Belkhir et al. 2001) using 500 permutations. For each sample, GENETIX calculated the number of loci pairs in which 5% and 1% of the permutations had a smaller value than the actual value for the sample. Linkage disequilibrium can be an indication that the sample contains family groups or the offspring of matings of genetically distinct populations, or that the population is under selection, or that alleles have drifted due to a small subpopulation size. Basic genetic diversity measures including gene diversity (Nei's (1987) estimate of heterozygosity corrected to minimum eight individuals) and allelic richness (average number of alleles per locus corrected to minimum eight individuals) were calculated using FSTAT. We used Wilcoxon signed-rank tests to test for significant differences among sample categories in gene diversity, allelic richness, and *N*_e_ that might signal effects associated with supplementation. Results for all tests were adjusted for multiple comparisons (sequential Bonferroni correction, Rice 1989) to an alpha level of 0.05.

Two methods were used to estimate *N*_e_; *N*_e_ is a fundamental parameter determining the evolutionary potential of a population, yet different techniques employ different assumptions and yield somewhat different results. Tracking changes in *N*_e_ may indicate changes associated with supplementation (Antao et al. 2010; Hare et al. 2011). We estimated *N*_e_ using linkage disequilibrium (LD-*N*_e_, Waples 2006) in the program LDNe (Waples and Do 2008; setting the lowest frequency allele at 2% to avoid bias introduced by small collections) and estimated *N*_e_ using a method based on maximum likelihood pairwise sibship analysis (SA-*N*_e_, Wang 2009) implemented in the program COLONY 2.0.0.1. Because chum salmon have overlapping year classes, the values from these measures estimated the number of breeders (*N*_b_) giving rise to the collection, rather than the actual *N*_e_. To calculate actual *N*_e_, the *N*_b_ is multiplied by the generation time, which is 3–5 years in chum salmon. Because generation time is variable in chum salmon (especially variable in these recovering subpopulations), we simply referred to the values calculated for a single-year mixed-age collection by LD and SA as *N*_e_ and did not multiply by generation time. Because assumptions and results varied somewhat between methods, we calculated a harmonic mean (and variance) of *N*_e_ values (Hm*N*_e_) from the two methods, following Waples and Do (2010)_._ We also calculated the ratio of Hm*N*_e_ to the census size for spawner types as a metric to investigate differences among sample types and changes over time (Hedrick 2005).

Effective population size analyses were conducted from a metapopulation perspective (Gomez-Uchida et al. 2013): we calculated the metapopulation *N*_e_ before supplementation for the earliest original program samples (00Union_O, 85Lilli_O, 99Hamma_O, 92Quil_O, 86Salmon_O and 86JCL_O) and after supplementation for the latest collected wild-born samples (08Union_W, 05_06Lilli_W, 08Hamma_W, 08_09Quil_W, 09Salmon_W, and 08_09JCL_W). The metapopulation *N*_e_ was first calculated according to the Stepping Stone model (Maruyama 1970) and the Island model (Wright 1943) using *N*_e_ values from the LD-*N*_e_ and SA-*N*_e_ and then we calculated the harmonic mean of the metapopulation LD-*N*_e_ and SA-*N*_e_ for final metapopulation Hm*N*_e_ values (meta-Hm*N*_e_) based on the Stepping Stone and Island models. For the Stepping Stone model the average immigration rate was estimated from the global *F*_ST_ values for the two sets of six collections (original and wild-born), and the same global *F*_ST_ values were used in denominators for the Island model.

### Subpopulation comparisons

A variety of measures assessed whether supplementation had induced changes in genetic attributes and genetic structure and whether strays from supplementation programs into unsupplemented tributaries had altered population structure or substructure. To assess possible changes in genetic attributes (heterozygosity, allelic richness, and Hm*N*_e_), we used Wilcoxon sign rank tests to test for significant differences in these genetic attributes among collection categories; original, supplementation hatchery-born, and wild-born (see Table 1). We tested for significant differences in genotypic distributions among all samples using GENEPOP3.3 (Raymond and Rousset 1995), and examined temporal and spatial partitioning of pairwise genetic variance with pairwise *F*_ST_ tests in GENETIX 4.03 (Belkhir et al. 2001). In pairwise *F*_ST_ tests we evaluated whether variance was significantly different from zero with 1000 permutations. A permutation test implemented in FSTAT (comparison among groups of samples) was used to test for significant differences in global *F*_ST_ values among categories of samples (original, supplementation hatchery-born and wild-born, see Table 1), with 10 000 permutations. To assess changes associated with supplementation, we compared pairwise *F*_ST_ values among original samples and among wild-born samples collected after supplementation programs had been initiated. These tests were conducted with and without samples from Dosewallips and Duckabush, the tributaries that were not deliberately supplemented. To assess changes in variance associated with hatchery supplementation programs, we compared pairwise *F*_ST_ values among samples of hatchery-born spawners and wild-born spawners collected in the same tributaries.

### Genetic distances

We assessed subpopulation structure using a principle coordinates analysis (PCoA) implemented in GenAlEx 6.5 (Peakall and Smouse 2006; Peakall and Smouse 2012). GenAlEx calculated pairwise *F*_ST_ values among populations over all loci and conducted a PCoA of the pairwise values. The PCoA is a multivariate ordination technique that describes underlying patterns in a dataset. The PCoA generates axes describing genetic variance in a dataset, here pairwise *F*_ST_ values, with the first two axes usually describing the maximum variance. As another means to view genetic relationships, we plotted Cavalli-Sforza and Edwards (1967) chord distances among collections in a dendrogram with 10 000 bootstrap replications using programs within PHYLIP (Felsenstein 1993).

### Assignment tests

To investigate whether fish sampled from the same tributary were more or less likely to be genetically similar to each other following supplementation, we used self-assignment tests in GeneClass2 (Piry et al. 2004). GeneClass employs the Rannala and Mountain (1997) algorithm in a ‘leave one out’ protocol and calculates the likelihood that an individual fish originated in the subpopulation in the tributary where it was sampled (home collection) based on the genotype of the fish and allele frequencies in the baseline collections, with the fish in question removed from its home collection in the baseline. There was no threshold likelihood value for assignment, the highest likelihood was accepted as the assignment and self-assignments were conducted on collections before and after supplementation. We tested for differences in percentage of assignments to home collection before and after supplementation using paired Student's *t-*tests.

### Isolation by distance

Data were compared before and after supplementation for evidence of changes in isolation by distance (IBD) patterns (Slatkin 1993), using transformed pairwise *F*_ST_ values [*F*_ST_/(1-*F*_ST_)] as a genetic similarity measure (Rousset 1997). Geographical distances (kilometers) between mouths of streams were calculated using the most direct passage over open water. Mantel tests for association between pairwise *F*_ST_ values and distance, and reduced major axis regressions were performed using IBD 3.23 (Bohonak 2002). To assess changes that might have occurred as a result of supplementation, we used ancova to compare IBD patterns among original collections to IBD patterns among collections of wild-born fish after supplementation began. We also compared IBD patterns of wild-born samples and hatchery-born samples to evaluate differences between the sample groups. We conducted analyses with and without Dosewallips and Duckabush collections because strays from hatchery programs were documented in spawner surveys in those tributaries and may have supplemented those populations.

## Results

### Supplementation and escapement

Supplementation programs boosted abundance of spawners and contributed from 5% to 96% of spawners to escapements in supplemented HC and SJF subpopulations (Table 2). The proportion of hatchery-born spawners decreased as successful programs terminated according to protocols (WDFW and PNPTT 2007). From 2001–2011, strays from supplementation programs contributed from 0% to 33% (average = 10%) of spawners to Dosewallips and Duckabush (Table 2), which were not deliberately supplemented. Most strays to Duckabush and Dosewallips were from nearby supplementation programs in Hamma Hamma and Quilcene (WDFW and PNPTT 2007). Similarly, most strays in other tributaries were from nearby supplementation programs and straying may be influenced by the natural exchange rate among these subpopulations.

### Subpopulation statistics

**Figure 2:**
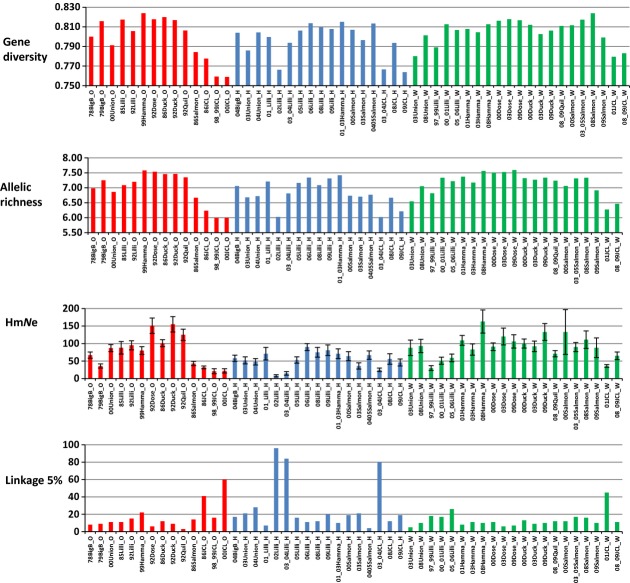
Plots of genetic statistics for samples grouped into original, hatchery, and wild categories. Genetic statistics are detailed in Tables 1 and 2.

Genotypic coverage averaged 95% and ranged from 84% (*One*-106) to 97% (*Oki*-1) per locus over all individuals (*N* = 2086). Because samples (tissues and archived scales) were from spawner carcasses of varying freshness, genotyping success varied among samples, regardless of contemporary or historical status. In tests for Hardy–Weinberg equilibrium (HWE) at individual loci (Supplementary Information, Table S3), two tests out of 832 were significant after corrections for multiple tests and all samples except 08Salmon_W were in HWE in tests over all loci (Table 1). The Wilcoxon sign rank tests indicated no significant differences between original and wild-born samples in genetic diversity measures (gene diversity, allelic richness) or Hm*N*_e_ (Fig. 2, Table 3, see Tables 1 and 4 for individual collection values). However, hatchery-born spawners had lower genetic diversity and significantly lower Hm*N*_e_ than wild-born spawners (differences in genetic diversity were not significant when Dosewallips and Duckabush were excluded from wild samples but differences in Hm*N*_e_ between wild-and hatchery-born samples remained significant).

**Table 3:** Table of averages (avg) and harmonic means (hmean) of genetic statistics for categories of samples (original = O, hatchery-born = H, wild-born = W) detailed in Tables 1 and 4, and *P* values for Wilcoxon signed-rank tests for comparisons

					No DoseDuck		
		O	W	*P* value	O	W	*P* value
Gene Diversity	avg	0.7996	0.8053	0.4332	0.7946	0.8027	0.2259
Allelic Richness	avg	6.98	7.15	0.2398	6.84	7.05	0.1819
Hm*N*_e_	hmean	52.41	76.99	0.1332	45.05	69.57	0.0629
Link 5%	hmean	10.00	10.72	0.5000	10.59	11.81	0.4896

		H	W	*P* value	H	W	*P* value
Gene Diversity	avg	0.7969	0.8053	0.0457	0.7969	0.8027	0.1774
Allelic Richness	avg	6.82	7.15	0.0060	6.82	7.05	0.0585
Hm*N*_e_	hmean	36.11	76.99	0.0002	36.11	69.57	0.0039
Link 5%	hmean	14.25	10.72	0.0192	14.25	11.81	0.0794

Analyses were conducted with and without Duckabush and Dosewallips samples (DoseDuck). Gene diversity is Nei's estimate of heterozygosity corrected to a sample size of 6, and allelic richness is average number of alleles per locus corrected to a samples size of 6.

**Table 4:** Population statistics for summer chum salmon collections including gene diversity (‘GeneDiv’, Nei's estimate of heterozygosity corrected to a sample size of 8) and allelic richness (‘A_R_’, average number of alleles per locus corrected to a sample size of 8)

Region	Name	GeneDiv	A_R_	Hm*N*_e_	±2SD	Escape	HmN_e_/N
HC	78BigB_O	0.7999	6.98	67	9	680	0.10
HC	79BigB_O	0.8158	7.25	36	6	191	0.19
HC	04BigB_H	0.8039	7.06	58	9	1916	0.03
HC	00Union_O	0.7913	6.86	87	10	744	0.12
HC	03Union_W	0.7801	6.55	88	22	7906	0.01
HC	03Union_H	0.7858	6.68	52	10	4010	0.01
HC	04Union_H	0.8043	6.72	48	9	2378	0.02
HC	08Union_W	0.8013	7.05	93	19	1043	0.09
HC	85Lilli_O	0.8174	7.09	88	18	92	0.96
HC	92Lilli_O	0.8056	7.20	95	13	99	0.96
HC	97_99Lilli_W	0.7891	6.82	30	7	64	0.47
HC	00_01Lilli_W	0.8126	7.33	51	10	63	0.81
HC	01_Lilli_H	0.7996	7.21	71	18	51	1.39
HC	02Lilli_H	0.7662	6.03	8	3	822	0.01
HC	03_04Lilli_H	0.7937	6.81	15	5	1207	0.01
HC	05_06Lilli_W	0.8068	7.22	59	11	685	0.09
HC	05Lilli_H	0.8062	7.16	53	9	790	0.07
HC	06Lilli_H	0.8136	7.34	90	10	1189	0.08
HC	08Lilli_H	0.8096	7.09	75	14	489	0.15
HC	09Lilli_H	0.8078	7.31	81	15	186	0.44
HC	99Hamma_O	0.8239	7.58	80	11	255	0.31
HC	01Hamma_W	0.8078	7.37	109	14	1155	0.09
HC	03Hamma_W	0.8045	7.17	83	16	536	0.15
HC	01_03Hamma_H	0.8151	7.42	71	14	390	0.18
HC	08Hamma_W	0.8127	7.56	163	33	1371	0.12
HC	92Dose_O	0.8177	7.54	151	22	655	0.23
HC	00Dose_W	0.8162	7.50	91	11	1260	0.07
HC	03Dose_W	0.8178	7.53	120	24	6510	0.02
HC	09Dose_W	0.8167	7.59	106	19	1093	0.10
HC	86Duck_O	0.8199	7.46	101	10	234	0.43
HC	92Duck_O	0.8169	7.46	155	22	617	0.25
HC	00Duck_W	0.8121	7.32	100	13	464	0.22
HC	03Duck_W	0.8026	7.27	92	15	1600	0.06
HC	09Duck_W	0.8062	7.34	133	24	2499	0.05
HC	92Quil_O	0.8063	7.35	125	16	743	0.17
HC	08_09Quil_W	0.8109	7.24	71	9	5353	0.01
SJF	86Salmon_O	0.7842	6.67	43	6	582	0.07
SJF	00Salmon_W	0.8116	7.06	133	64	439	0.30
SJF	00Salmon_H	0.8069	6.74	64	13	407	0.16
SJF	03_05Salmon_W	0.8173	7.31	90	13	7642	0.01
SJF	03Salmon_H	0.7965	6.70	36	9	1866	0.02
SJF	04_05Salmon_H	0.8134	6.77	67	12	4203	0.02
SJF	08Salmon_W	0.8239	7.33	111	25	1544	0.07
SJF	09Salmon_W	0.7991	6.92	88	28	1218	0.07
SJF	86JCL_O	0.7776	6.23	32	4	292	0.11
SJF	98_99JCL_O	0.7593	5.99	21	7	105	0.20
SJF	00JCL_O	0.7589	6.00	22	6	55	0.40
SJF	01JCL_W	0.7795	6.27	36	4	251	0.14
SJF	03_04JCL_H	0.7665	6.02	25	5	1427	0.02
SJF	08_09JCL_W	0.7831	6.46	65	11	1099	0.06
SJF	08JCL_H	0.7936	6.67	56	15	481	0.12
SJF	09JCL_H	0.7638	6.21	46	10	2102	0.02

Harmonic mean N_e_ (Hm*N*_e_) was calculated for *N*_e_'s from linkage disequilibrium (LD-*N*_e_) and pairwise sibship analysis (SA-*N*_e_). Escapement (Escape) was calculated using area under the curve and fish counts at traps and sums wild-and supplementation hatchery-origin escapements to natural spawning grounds. The Hm*N*_e_/N is the ratio of Hm*N*_e_ to escapement.

Linkage disequilibrium and pairwise sibship analyses suggested most collections included some family groups, ranging from a pair of siblings to large families (data not shown). There were 120 locus pairs examined per collection, and most collections had six or more locus pairs in linkage disequilibrium at the 5% level and 12 collections had six or more locus pairs in linkage disequilibrium at the 1% level (Table 1). The Wilcoxon signed-rank test indicated that hatchery-born spawners had significantly more locus pairs in linkage disequilibrium than wild-born spawners (*P* = 0.019). The collections from 02Lilli_H, 03_04Lilli_H, and 03_04JCL_H were notable for high linkage disequilibrium: COLONY calculated a single family with 14 full-siblings in 02Lilli_H (12% of pairwise relationships were full-siblings), seven families with between three and six full-siblings in 03_04Lilli_H, and six families with between three and eight full-siblings in 03_04JCL_H (4% of pairwise relationships were full-siblings). Siblings in collections were noted but not removed.

### Effective population size calculations

**Figure 3:**
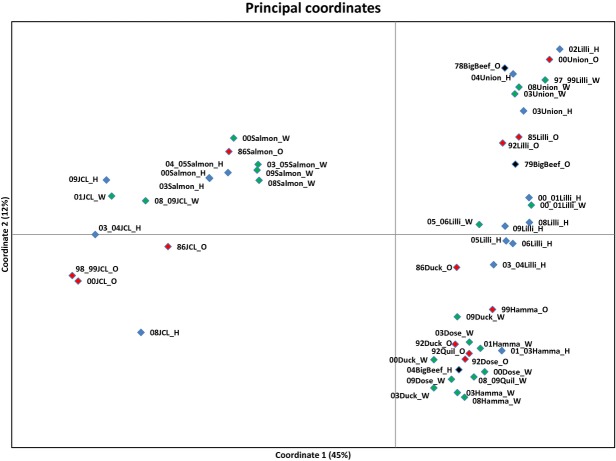
Principle coordinates plot of pairwise *F*_ST_ values among summer chum salmon collections from Hood Canal and Strait of Juan de Fuca. Name abbreviations follow Table 1 and colors for categories follow Fig. 2, with the exception of Big Beef (in black): ‘78 and 79 Big Beef’ collections were sampled prior to extinction, and the ‘04 Big Beef’ collection was derived from Quilcene (Quil) broodstock.

We calculated *N*_e_ and its 95% parametric confidence interval for each collection with two methods: linkage disequilibrium (LD-*N*_e_) and pairwise sibship analysis (SA-*N*_e_). In most calculations, the two values were similar and the 95% confidence intervals overlapped (see data for both methods for each collection in Supplementary Information Table S4 ‘All_N_e_’). Differences arose for collections with high linkage and small samples sizes, which biased LD-*N*_e_ downward (Wang and Whitlock 2003; Waples and Gaggiotti 2006), and where data were mostly lacking for a single locus, which depressed SA-*N*_e_ in 79Big Beef sample. The harmonic means of *N*_e_ values (Hm*N*_e_) varied over time and space, both within and between tributaries (Table 4, Fig. 3). Where there were samples from hatchery-born and wild-born spawners collected in the same tributary in the same year, the hatchery-born samples generally had a smaller Hm*N*_e_ (e.g. 03Union_H and 03Union_W), except for samples from Lilliwaup Creek: in two comparisons of hatchery-and wild-born samples for that creek, the Hm*N*_e_ for the hatchery-born samples was equal to or greater than the Hm*N*_e_ for the wild-born sample. In the original Lilliwaup samples, the Hm*N*_e_ values were similar to those of Union River, but they declined to less than half by 1997. We lack wild-born samples from Lilliwaup Creek after 2006, but in the most recent hatchery-born samples, the Hm*N*_e_ values were similar to original values, suggesting that diversity may be recovering in the Lilliwaup Creek subpopulation. The Hm*N*_e_ in the original Jimmycomelately Creek sample was roughly half the value of the most recent wild sample, suggesting that diversity is also recovering in the Jimmycomelately Creek subpopulation. The calculated ratios of Hm*N*_e_ to census size (Hm*N*_e_/N in Table 4) were significantly lower in wild-born samples than in original samples (Wilcoxon sign rank test, *P* = 0.0011 and *P* = 0.0137 with Dosewallips and Duckabush collections included and excluded, respectively), likely reflecting increases in census size (N) throughout the restoration program. Further, in an anova the ratio of Hm*N*_e_ to census size for the wild-born fish was negatively correlated with the number of years of supplementation (*F*_1,68_ = 11.2, *P* = 0.001).

The global *F*_ST_ values for the six original and six wild-born samples were 0.029 and 0.021, respectively (*P* = 0.2414), resulting in calculated immigration rates of 8.37 and 11.65, respectively. The meta-Hm*N*_e_ for the original and wild-born samples was 488 and 591, respectively, with the Stepping Stone model, and 502 and 603, respectively, with the Island model (see Supplemental Information Table S4 for LD-*N*_e_ and SA-*N*_e_ values of six original and six wild-born samples and calculations for meta-Hm*N*_e_ values). Paired *t*-tests for LD-*N*_e_ and SA-*N*_e_ values indicated no significant differences between values for original and wild-born fish (*P* = 0.1873 for LD-*N*_e_ values for original versus wild-born fish, *P* = 0.3697 for SA-*N*_e_ values for original versus wild-born fish). Although meta-Hm*N*_e_ increased following supplementation, the difference was not significant.

In one further consideration of *N*_e_, we computed the harmonic mean of the calculated *N*_e_ values over all collections with hatchery-born and wild-born spawners combined in collections and separated (see Supplementary Information, Table S5 ‘All_HmNe’). The calculated value for the wild-born spawners (78.95 ± 33.86) was larger than the value calculated for the hatchery-and wild-born spawners combined (55.19 ± 37.44), but the confidence intervals overlapped. Also, within some single-year examinations, the ratios of Hm*N*_e_/N were higher in uncombined than in combined collections, suggesting Ryman–Laikre effects from combining hatchery-and wild-born fish into single collections.

### Genetic variance patterns within and between subpopulations

Pairwise genotypic and *F*_ST_ tests were mostly congruent (see Supplementary Information Table S6 for pairwise test values and their associated *P* values). Pairwise tests indicated temporal stability within most subpopulations, with the exception of Lilliwaup Creek (see Fig. 3 for a plot of pairwise *F*_ST_ values along principle coordinate axes). Among Lilliwaup Creek samples, 37 of 66 pairwise genotypic comparisons were significant and 34 of 66 pairwise *F*_ST_ comparisons were significant. Union River samples also differed from all other HC samples and are the most isolated geographically (Fig. 1). There was little differentiation among samples from Dosewallips, Duckabush, Hamma Hamma, and Quilcene rivers. The SJF samples differed from each other and were distinct from HC samples. The original 1970s Big Beef Creek samples were most similar to those of Union River and Lilliwaup Creek, and the reintroduced sample was most similar to the sample from Quilcene River, its broodstock source, and consequently similar to Dosewallips, Duckabush, and Hamma Hamma rivers samples (Fig. 3).

Global *F*_ST_ values for different categories of samples did not change significantly throughout the program (Table 5). Although the global *F*_ST_ value for wild-born samples was lower than the value for original samples, the decrease following supplementation was not significant. The global *F*_ST_ for original samples matched the global *F*_ST_ for the hatchery samples, suggesting that the hatchery programs captured similar genetic variance among subpopulations. Including original and wild-born collections from Dosewallips and Duckabush in computations lowered global *F*_ST_ values, but changes were still not significant.

**Table 5:** Table of global *F*_ST_ values for categories of samples and *P* values for permutation test comparison among category values

	*N* samples	Global *F*_ST_	*P* value
Original, all samples	12	0.024	0.294
Wild, all samples	21	0.020	
Original, no Dose no Duck	9	0.032	0.122
Wild, no Dose no Duck	15	0.021	
Original, only Dose and Duck	3	0.001	0.522
Wild, only Dose and Duck	6	0.003	
Hatchery	16	0.032	0.098
Wild, no Dose no Duck	15	0.021	

The ‘N samples’ are the number of samples in the category. Big Beef Creek samples were excluded because the program was a reintroduction.

### Genetic clusters identified in PCoA and dendrogram

**Figure 4:**
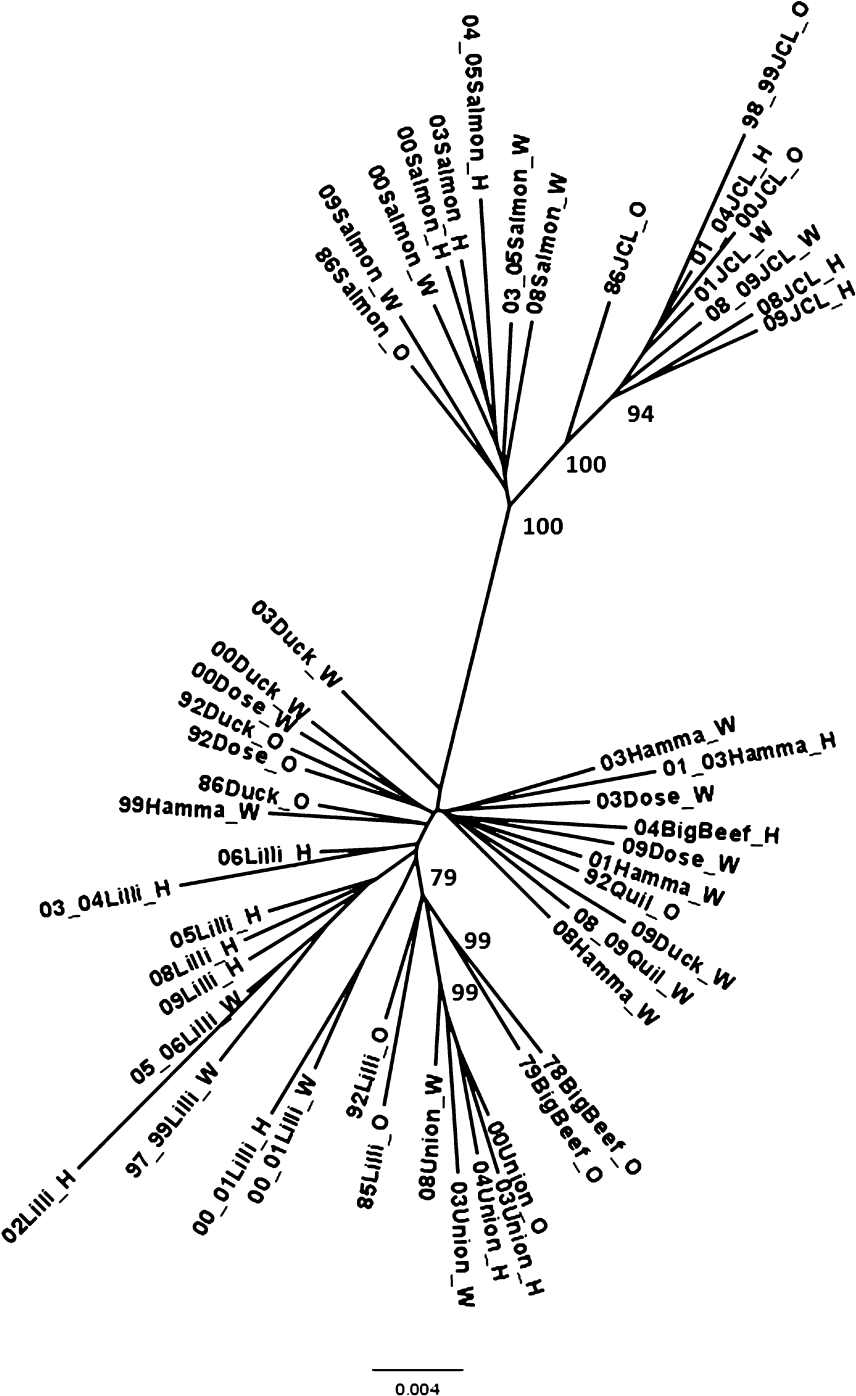
Neighbor-joining dendrogram of Cavalli-Sforza and Edwards chord distances among samples. Bootstrap values over 70% are plotted on the tree nodes.

Population centers formed two major clusters in the principle coordinates analysis plot (Fig. 3), and there was no distinction detected between hatchery and wild samples from the same tributaries. The first axis explained 45% of the genetic variance: SJF samples clustered on the left, and HC samples clustered on the right. The second axis explained 12% of the variance: The HC populations divided loosely into two to three clusters along the second axis. The lowest cluster included samples from Dosewallips, Duckabush, Hamma Hamma, and Quilcene rivers as well as the 2004 collection from Big Beef Creek. The topmost cluster included the original samples from Big Beef Creek (these separated along the third axis; the 1979 collection was missing most of one locus and plotted distantly from the 1978 collection), Union River samples, and the older samples from Lilliwaup Creek. The more recent Lilliwaup samples clustered mainly in the center, supporting high genetic drift in this population. The placement of the original Big Beef samples indicated that before reintroduction, summer chum salmon in Big Beef Creek were genetically more similar to other subpopulations from low-elevation tributaries on the east side of HC rather than the geographically closer subpopulations in tributaries originating in the Olympic Mountains (Dosewallips and Duckabush) on the west side of HC. A dendrogram of genetic distances among samples (Fig. 4) displayed similar overall structure. The SJF and HC clusters separated with 100% bootstrap support, with JCL forming a supported sub-branch. The Dosewallips, Duckabush, Hamma Hamma, and Quilcene samples formed an unsupported cluster, and the original samples from Lilliwaup Creek clustered with 79% bootstrap support on a branch with Union River and the original Big Beef Creek samples. The remaining Lilliwaup Creek samples clustered on three unsupported branches. There were no distinctions evident between hatchery-born and wild-born samples collected in the same tributaries.

### Assignment test

Assignment tests supported a metapopulation structure with straying among close subpopulations, especially along the west side of HC (Dosewallips, Duckabush, Hamma Hamma, and Quilcene, Table 6). Where high gene flow occurs, an individual may be equally likely to have been sampled in two or more baseline populations or may assign to a closely related population. In presupplementation collections, self-assignments were low to indistinguishable from random in Dosewallips, Duckabush, Hamma Hamma, and Quilcene rivers. If we combined results from these rivers, assignments back to the combined ‘HoodMet’ were high (Table 6). Self-assignments were relatively high for Salmon and Jimmycomelately creeks and moderate in Union, Lilliwaup, and Big Beef creeks. Following supplementation, self-assignments decreased in all but Union River and Hamma Hamma River, but the decrease was not significant (Student's paired *t*-test, *P* = 0.054).

**Table 6:** Table of assignments using GeneClass2

	Presupplementation assignments										
	78_79Big Beef_W	00Union_W	92Lilli_W	92Dose_W	92Duck_W	99Hamma_W	92Quil_W	86Salmon_W	00JCL_W	Total	
78_79Big Beef	**55**	4	11	3	7	4	2	1	0	87	
00Union	11	**35**	0	1	1	2	3	1	0	54	
92Lilli	9	3	**20**	2	4	4	4	0	0	46	
92Dose	6	1	3	**15**	13	5	5	0	0	48	
92Duck	1	0	3	15	**16**	5	5	1	0	46	
99Hamma	5	2	3	8	8	**4**	3	1	0	34	
92Quil	3	0	3	10	4	10	**20**	0	0	50	
86Salmon	2	0	0	3	2	0	1	**31**	3	42	
00JCL	0	0	0	0	0	1	1	3	**49**	54	
											HoodMet
Correct	55	35	30	15	16	4	20	31	49		146
Total	87	54	46	48	46	34	50	42	54		178
% correct	63.22	64.81	65.22	31.25	34.78	11.76	40.00	73.81	90.74		82.02

	Postsupplementation assignments										
	04BigBeef_H*	08Union_W	09Lilli_H	09Dose_W	09Duck_W	08Hamma_W	08_09Quil_W	09Salmon_W	08_09JCL_W	Total	
04BigBeef_H*	**22**	1	3	8	3	4	4	0	0	45	
08Union_W	3	**35**	3	1	2	3	0	0	0	47	
09Lilli_H	5	4	**11**	1	3	1	4	0	0	29	
09Dose_W	6	0	3	**4**	4	10	6	0	0	33	
09Duck_W	4	1	2	5	**9**	7	6	0	0	34	
08Hamma_W	4	2	4	6	3	**10**	12	0	0	41	
08_09Quil_W	6	3	1	5	5	11	**14**	0	0	45	
09Salmon_W	0	0	0	0	0	1	0	**16**	6	23	
08_09JCL_W	0	2	0	0	0	0	0	4	**27**	33	
											HoodMet
Correct	22	35	11	4	9	10	14	16	27		117
Total	45	47	29	33	34	41	45	23	33		153
% correct	48.89	74.47	37.93	12.12	26.47	24.39	31.11	69.57	81.82		76.47

Abbreviations follow Table 1. Assignments for each collection are along rows with total fish analyzed in collection at side and bottom. Assignments back to collection of origin (‘correct’ assignments) are in bold type along diagonal. The ‘% correct’ was ‘correct’ assignments over total assigned. The category ‘HoodMet’ sums the number of assignments back to the Hood Canal ‘metapopulation’ group encompassing results for Dosewallips, Duckabush, Hamma Hamma, and Quilcene.

* Reintroduced using Quilcene broodstock.

### Isolation by distance

**Figure 5:**
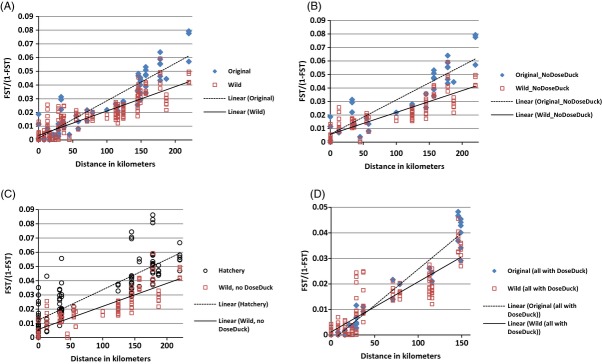
Plots of isolation by distance patterns (transformed pairwise *F*_ST_ value (*F*_ST_/(1 −* F*_ST_) versus geographical distance) for different categories of samples: plot (A) all original samples and all wild samples, plot (B) original and wild samples (minus Dosewallips and Duckabush), plot (C) hatchery and wild samples (minus Dosewallips and Duckabush), and plot (D) only original and wild samples in comparison with Dosewallips and Duckabush. Plot (A) includes data from plots (B and D).

We conducted IBD analyses for all categories of samples (Fig. 5A–D), both including and excluding Dosewallips and Duckabush samples. In all analyses, physical distance explained a significant amount (all *r*^2^ > 70%) of the genetic variance among samples (all *P* < 0.001). In comparisons among sample categories, because the tests for homogeneity among regressions in the ancova indicated significant differences between regressions, we were unable to compare regressions statistically. However, the IBD slope was steeper for comparisons among original samples (Fig. 5A,B) than the slope for comparisons among wild samples, suggesting that genetic variance among populations decreased with supplementation. The IBD slopes were similar for comparisons among hatchery-born and wild-born samples (Fig. 5C), but genetic distances were greater among hatchery-born fish. When analyses were limited to comparisons involving Dosewallips and Duckabush samples (Fig. 5D), the slope for the original samples was slightly steeper than the slope for the wild samples.

## Discussion

This study joins a growing body of research on the genetic effects of supplementation hatchery programs on wild fish populations. We examined a time series of a summer chum salmon metapopulation (a designated ESU) from before, during, and after supplementation, providing a temporal perspective of supplementation and responses by threatened subpopulations. We found that after several years of hatchery supplementation, there was little change in genetic diversity and harmonic means of effective population sizes (Hm*N*_e_) in wild-born salmon returning to spawning areas throughout the ESU. However, genetic distances within the metapopulation decreased and assignments back to collection of origin decreased following supplementation, suggesting higher gene flow within the metapopulation and lower genetic drift in subpopulations. Hatchery-born spawners collected in the same locations as wild-born spawners usually had lower genetic diversity and smaller Hm*N*_e_ values, suggesting that supplementation hatchery programs sampled a subset of genetic diversity in the target subpopulations and that factorial matings of hatchery broodstocks may have decreased Hm*N*_e_. Higher linkage disequilibrium in hatchery-born fish indicated potential for Ryman–Laikre effect, and combining hatchery-and wild-born fish into single collections lowered ratios of effective population sizes to census sizes. However, with the exception of the Lilliwaup Creek subpopulation, suspected Ryman–Laikre effects did not increase genetic drift in wild-born spawners. Thus, possible negative impacts of supplementation appeared minimal and likely diminished as wild-born spawner abundance increased, suggesting that population recovery in this summer chum salmon ESU was unimpaired by supplementation.

### Supplementation and conservation

In the Pacific Northwest, supplementation hatcheries using local broodstocks have received increased interest as a tool in fish conservation to preserve native genetic diversity and maintain a foundation of response to environmental variation. Traditional fish hatcheries were a mainstay of fish management and harvest augmentation, but they introduced problems when nonlocal, hatchery-adapted fish interacted with wild fish (Araki et al. 2008; Christie et al. 2012). Supplementation hatcheries were designed to reduce problems associated with traditional hatcheries in that domestication selection was minimized by using in-river broodstocks originally composed of wild-origin fish (subsequent broodstocks would be mixtures of hatchery-and wild-origin fish) and by reforming hatchery practices. Hatchery fish from the same gene pool as wild fish were expected to be similarly adapted to the local environment. Here we discuss studies documenting impacts from supplementation programs and hypothesizing mechanisms leading to differences between hatchery and wild fish in relation to chum salmon.

One selective force in supplementation hatchery programs is juvenile residence in a hatchery environment (McClure et al. 2008). Hatchery effects may be relatively lower for chum salmon because they out-migrate shortly after emergence, spending minimal time under hatchery rearing conditions. In contrast, most supplementation studies have been conducted in species with extended juvenile freshwater residence times [steelhead, Atlantic salmon (*Salmo salar*), coho and Chinook salmon (reviewed by Araki et al. 2008 and Chilcote et al. 2011)]. In these species, juvenile residence time in freshwater is a critical component of their life history, and unless they are released as unfed fry, hatchery-origin juveniles often spend a minimum of 1 year in a hatchery environment before out-migrating. Kostow (2004) observed differences in steelhead juvenile phenotypes and lower survival in hatchery-origin juveniles from the same parent pool. In a common garden experiment, Chittenden et al. (2011) found that for coho salmon juvenile rearing environment was a key influence on smolt size and multiple behaviors, regardless of ancestry. Thériault et al. (2011) implicated sexual selection at the juvenile stage, especially on males, as a factor decreasing relative reproductive success (RRS) in hatchery-origin coho salmon spawning in the wild.

Another selective force in supplementation hatchery programs is human-controlled spawning (McClure et al. 2008), and chum salmon would be impacted similar to other species. Artificial spawning bypasses mate selection, and Consuegra and Garcia de Leaniz (2008) found a positive association between disassortative mating (natural spawners choose mates with dissimilar MHC profiles) and parasite resistance: In Atlantic salmon offspring parasite load decreased their RRS. Further, the summer chum salmon supplementation programs used factorial matings, which likely decreased effective population size relative to single-pair matings (Abadia-Cardoso et al. 2013). Multiple factors likely contribute to the phenomenon that hatchery-origin fish and wild-origin fish with hatchery ancestry are less productive under natural spawning conditions (Araki et al. 2008; Chilcote et al. 2011).

In other studies, supplementation impacts were undetected or equivocal. In Berejikian et al.'s (2009) study, hatchery-and wild-born summer chum salmon spawners (Quilcene fish that were included in this study) mated randomly and there was no significant difference in offspring produced per spawner type. Sharpe et al. (2010) found no significant difference in reproductive success of wild-origin and first-generation hatchery-origin steelhead derived from the same gene pool. Further, genetic diversity in steelhead populations supplemented with native broodstock remained unchanged after 20 years (Heggenes et al. 2006) and 58 years (Gow et al. 2011). There was also no loss in genetic diversity (Eldridge and Killebrew 2008) or fitness (Hess et al. 2012) in Chinook salmon supplemented with native broodstock. In this study, we lack parentage data to address RRS (see Christie et al. 2012) and address only changes in genetic parameters. Yet, coupled with the study by Berejikian et al. (2009), the increases in wild-born spawner abundance and increases in recruits per spawner suggested that hatchery-born HC summer chum salmon spawned successfully, which may have contributed to increased census sizes and lower extinction risks and contributed to recovery in the metapopulation (Wang and Ryman 2001). The lower Hm*N*_e_/N ratio in wild-born spawners could also be an indication that some of their parents were hatchery-born fish that, although abundant, had lower genetic diversity than wild-born fish because of unequal hatchery family sizes.

### Metapopulation structure and supplementation

Contemporary genetic structure in HC summer chum salmon follows an IBD pattern, similar to original genetic patterns (Phelps et al. 1994; Small et al. 2009), in which the amount of genetic exchange depended on distance between spawner groups (Schtickzelle and Quinn 2007). Interestingly, the original collections from Big Beef Creek were most similar genetically to the collection from Union River, which is roughly 80 km distant, rather than to the closest tributary, Dosewallips River, roughly 8 km west across HC (see Fig. 1). This supports an ecoregional association between summer chum salmon inhabiting lowland streams on the Kitsap Peninsula on the east side of HC as suggested by Sands et al. (2009).

The relationship between genetic distance and geographical distance changed following supplementation in this summer chum salmon metapopulation – gene flow increased and genetic distances decreased – but the basic IBD pattern remained consistent over time. In another supplementation program for coho salmon, genetic distances increased following supplementation, which was attributed to genetic drift, bottlenecks, and varying success of between-river transfers (Eldridge et al. 2009). In HC summer chum salmon, each supplementation program used river-specific broodstocks rather than a common broodstock for all rivers. Thus, between-river transfers would occur via strays (nonhoming), which are documented in WDFW and PNPTC (2007). As a byproduct of increasing abundance, supplementation may have increased straying and decreased differentiation or perhaps restored straying to levels that existed prior to supplementation when subpopulations were more abundant. The pairwise *F*_ST_ values and assignment tests support that subpopulations are well connected through gene flow, which may have contributed to the increases in Hm*N*_e_ values. Alternatively, prior to supplementation census sizes were low and genetic drift may have increased differentiation; following supplementation and the fishing moratorium, increased census sizes may have countered genetic drift and decreased differentiation.

In summary, our results show that, although changes were not significant, in the HC summer chum salmon ESU contemporary genetic diversity and effective population sizes increased from original levels, suggesting that as natural production increased, possible negative impacts from supplementation (such as depressed *N*_e_ from Ryman–Laikre effects) diminished. We suspect that negative genetic impacts may be less or shorter-lived for chum salmon because their juveniles spend little time in the hatchery environment. We will continue to monitor this metapopulation to assess the long-term impacts of supplementation and success of recovery efforts.

## Data archiving statement

Raw data for this manuscript are available in the Dryad Digital Repository: http://dx.doi.org/10.5061/dryad.k2q95.

## Supporting Information

**Table S1.** Details of all broodstocks including numbers of fish, sex ratio, proportion of natural spawners used in hatchery program, and number of fry released per brood year. **Table S2.** Years when supplementation programs were initiated and terminated and the last year of expected supplementation returns. **Table S3.** Details of genetic statistics per locus and per population, including gene diversity, richness, HWE and *P*-values. **Table S4.** Data and calculations for meta-LDNe and meta-SANe and meta-Hmean. **Table S5.** Data and calculations for HmNe with hatchery-and wildborn samples separate and combined within collection year and tributary. **Table S6.** Pairwise genotypic test results from FSTAT. **Table S7.** Pairwise FST values and the number of permuted values that were larger than the actual value.
